# Observations on the Interior Structure and Economy of the Conglobate Glands

**Published:** 1827-02

**Authors:** John Charles Ogilvie


THE LONDON

### Medical and Physical Journal.

N° 336, VOL. LVII.] FEBRUARY, 1827. [N° 8, *New Series*.

For many fortunate discoveries in medicine, and for the detection of numerous errors, the world is indebted to the rapid circulation of Monthly Journals; and there never existed any work, to which the Faculty, in Europe and America, were under deeper obligations, than to the Medical and Physical Journal of London, now forming a long, but an invaluable, series.—RUSH.

### ORIGINAL PAPERS,
AND
### CASES OBTAINED FROM PUBLIC INSTITUTIONS AND OTHER AUTHENTIC SOURCES.

### CONGLOBATE GLANDS.

*Observations on the Interior Structure and Economy of the Conglobate Glands.* By JOHN CHARLES OGILVIE, M.D.

As to the distribution and the exterior form and structure of the conglobate glands, there seems to be little, if any, difference of opinion, and our ordinary descriptions of them may therefore be confided in as sufficiently accurate. They are likewise as full and minute as most other parts of anatomy. With respect to the interior structure of these glands, however, and the particular functions which they may be supposed to perform in the animal economy, the case is different: of these our knowledge is very limited. Considerable differences of opinion have always existed, even among anatomists and physiologists of the first character; and it appears to me that the few facts which have been ascertained and generally admitted, have not met with that attention which they deserve, as illustrative both of physiology and pathology, as well as of the operation of medicines on the animal economy.

As far as I can learn the present state of opinion on this subject, all seem to allow that the vasa inferentia,—i. e. the lacteal or lymphatic vessels which carry the chyle or lymph into the glands, are divided and subdivided to an extreme degree of minuteness, as they pervade the substance of the glands; but there seems to be a wide difference of opinion as to the manner in which these minute ramifications of the vasa inferentia are ultimately disposed of. Some contend that the gland is made up entirely of convolutions of lymphatic vessels, intermixed with the minute ramifications of arteries, veins, and nerves; and that these are merely connected or bound together by common cellular membrane. The advocates of this opinion seem to think that the vasa inferentia, after dividing to a great extent of minuteness, and thus forming many convolutions, again unite into larger and larger branches, to form what are called the vasa efferentia,—viz. those lacteal or lymphatic vessels which carry the chyle or lymph from the gland, on its route towards the sanguiferous system. Others again maintain, that, besides this minute division and subdivision of the lacteal and lymphatic vessels in the substance of the glands, and their connexion with the still more minute ramifications of arteries, veins, and nerves, there is likewise a peculiar cellular structure,—i. e. a congeries of cells, proper or peculiar to these glands, and quite distinct and different from the common cellular substance by which this and all the other textures of the gland, and of the whole body, are bound together. Into these distinct and peculiar cells the extremities of the vasa inferentia are supposed to open and pour their contents; and from the same cells these fluids are supposed to be again absorbed by the minute beginnings of the vasa efferentia.

The latter of these opinions seems to have been for many years by much the more prevalent; and that there is such a cellular structure as I have just now mentioned, has been proved both by the late Mr. Cruickshank, in his work on the Absorbent System, and by Mr. Abernethy, in a valuable paper in the Philosophical Transactions for 1795. The former observes, that, when a gland is completely injected, it appears to consist merely of convolutions of vessels; but that, when it is about half filled with mercury, thrown in through the vasa inferentia, the cells are often very evident. When the injection is pushed far, the mercury is forced into the numerous ramifications of vessels which circulate on the parieties of the proper cells, and in the common cellular substance of the gland. When a gland is examined in this state, the numerous convolutions of vessels in a manner hide the cells from our view; and, even when the cells are seen, they are so covered with injected vessels as to be readily mistaken for mere convolutions of vessels. Mr. Cruickshank mentions, however, that he had injected many glands in which there was not the least appearance of a convoluted vessel; but adds, that he never injected one in which he did not see some cells, especially if he was attentive to the mercury just as it entered the gland. " In quadrupeds," he observes, " it is very easy to demonstrate this cellular texture."*

Mr. Abernethy injected the lymphatic glands of the axilla and groin of a horse with wax, and then destroyed the animal matter by immersing it in muriatic acid. In some of the glands the wax appeared in very small portions, and irregularly conjoined; which, he remarks, is a convincing proof that it had acquired this irregular form by having been impelled into numerous minute cells In various other instances, he found one solid lump of wax after the destruction of the animal substance; and he was satisfied that the glands which were filled in this manner were formed internally of one cavity, and were not, as is commonly the case, composed of many minute cells. He likewise filled glands of this structure in the mesentery of the horse with quicksilver; then dried them, cut open the bags, and introduced a bristle into them through the vas inferens. Also, in the human mesentery, after having injected the artery, he filled a bag, resembling a gland, with quicksilver; which, on being opened afterwards, was found to contain a mixture of injection and quicksilver,—i. e. a mixture of the injection that was thrown in through the artery, and of the quicksilver thrown in through the lacteal. In the mesenteric glands of the whale, Mr. A. found bags as large as an orange, and distinctly saw both arteries and veins, as well as lacteals, terminating in them by open mouths. The lacteals he mentions as opening into every part of them, and in great numbers. When quicksilver was poured into any of the lacteals which were found near the sides of the bags, it immediately ran in a stream into their cavities. He introduced about a dozen bristles, through as many lacteals, into different parts of two of these bags. These, he observes, were doubtless few in comparison to the whole number which terminated in them; but, as the mesentery was fat, and the vessels small, more could not easily be passed.†

These are but a few of the facts and arguments that have been adduced to prove the cellular structure of the conglobate glands; yet they appear sufficient to show that the glands in question contain certain proper cavities, with which certain vessels communicate by open mouths. These glandular cavities seem in most animals, with whose anatomy

* Page 85.

† Phil. Trans. 1796, lxxxvi.—Abridg. xvii. p. 673.

we are acquainted, to consist of a congeries of minute cells, communicating readily and freely with each other; but, from Mr. Abernethy's paper, it is evident that they vary greatly, both as to the number and size of the cells or sacs, in different species of animals, and sometimes in the same species, or even in the same individual. Thus, in the human species, they consist generally, or almost always, of a great number of cells, so very minute as to be distinguishable only with the microscope: while, in the whale, a gland is found to consist of a single bag, sometimes as large as an orange; and a similar organisation is occasionally met with in other animals, —sometimes even in the human subject. Whatever be the size or number of these bags or cells, they are evidently distinct cavities, and not, as some would seem to have supposed, a part of that cellular substance which is common to the whole body, and which enters as a constituent part into the composition of every organ in it. The common cellular substance enters, indeed, into the composition of these glands, as it does into that of every other part of the body; but it is quite distinct from the peculiar apparatus of cells which I have just now mentioned. The two textures are as distinct from each other as the air-cells of the lungs, the pelvis of the kidney, or, in short, the proper cavity of any viscus, is from the interstices of the common cellular substance which pervades every part of the viscus, and connects all its parts together.

Admitting now, as I think we must do, that the conglobate glands contain certain proper cavities, in which the lacteals and lymphatics terminate by open mouths, we must suppose that these vessels pour their contents into the cavities in question: nor can we doubt of the purpose for which they are made to do so. We can scarcely suppose that the chyle passes into the mass of blood in the same state in which it leaves the intestines. We must suppose something more than the transient action of the gastric and intestinal fluids necessary for the conversion of dead and comparatively gross matter into blood,—into that wonderful fluid, which is not only possessed of life itself, but which diffuses life and vigour through every part of the body. The chyle is considered by physiologists as possessed of a certain degree of vitality, even in the first stages of its formation, before it is taken up from the intestines; and, if certain kinds of dead animal and vegetable matter may acquire even the lowest degree of vitality from the action of certain secreted fluids in the stomach and bowels, it requires no great stretch of analogy to conclude that the vitality thus originally acquired may be essentially increased or exalted by the action of another secreted fluid in the cells of the mesenteric glands. That the cells of the conglobate glands do secrete a peculiar liquor, seems highly probable from all we know of their economy,—from the plentiful supply of blood with which they are uniformly furnished,—from the numerous terminations of arteries which Mr. Abernethy discovered opening into the sacs in the mesenteric glands of the whale,—from the slimy reddish fluid which he found in these sacs,—and from all the accounts that have yet been given of the fluid known by the name of "succus proprius glandularum."

It is more than probable that the chyle undergoes some very important change in this cellular structure, which I have represented as proper to the mesenteric and other conglobate glands; and analogy seems fully to warrant the inference that a corresponding change is effected on the lymph in the analogous structure of those glands through which it passes. This inference seems equally admissible, whether we consider the lymphatics as mere absorbents, or as deriving their origin, in part or in whole, from continuity with the arteries. If they be mere absorbents, some such process as I have supposed to go on in the glands seems the only means we have of accounting for the peculiar and uniform character which the lymph is found to exhibit in all parts of the body, and under almost all circumstances. If, on the other hand, we suppose the lymph to be immediately derived from the blood, we can only account for its temporary separation on the supposition of its being destined to undergo some important change before it be again mixed with the general circulating mass; and this supposed action of the glands seems to afford the only probable means of effecting such a change.

All that I have yet advanced seems in unison with the opinion I formerly mentioned as the prevailing one on this subject,—viz. that the chyle and lymph are poured into the cells of the glands by the extremities of the vasa inferentia; are taken up from thence by the minute beginnings, or radules, of the vasa efferentia; and carried by these vessels either directly to the thoracic duct, or into other glands which they are supposed to pervade in the same way as they do the first. Yet I cannot assent to this opinion in all particulars. While I consider it as undeniably proved that both chyle and lymph are poured into the glandular cavities, I think we have reason to believe that this is not the case with the whole of these fluids. I am convinced, on the contrary, that a considerable proportion of each pervades the gland without being effused from its vessels; and, of the portion which is effused into the cells, or other glandular cavities, by the extremities of the vasa inferentia, I doubt much whether any part finds its way back into the lacteal or lymphatic vessels. That a part of these fluids pervades the glands without being poured into their proper cavities, seems more than probable from what Mr. Abernethy observed in the mesenteric glands of the whale. The appearance I allude to is so remarkable, and seems so characteristic of the economy of these glands, that I must give it in his own words.

"Having removed the injection from these bags, I observed, on the inside of them, a soft whitish substance, apparently containing a plexus of lacteal vessels. This substance entered the bags at that part of them which was nearest to the intestines, and went out at the point next to the spine. I now poured some quicksilver into those lacteals which appeared to lead to this soft substance. The quicksilver soon entered the vessels which were contained in it, and thus its nature was ascertained. A number of lacteals having entered one of these bags, were observed to communicate with each other; then again to separate, and form other vessels which went out of the bag. It was some time before the quicksilver passed through the plexus of vessels contained in the bag, but, after having pervaded it, it passed on to a second bag, in which was concealed a similar plexus of lacteals. The quicksilver permeated these last vessels with much greater facility than it did the former, and quickly ran out at the large lacteals which were divided at the origin of the mesentery."*

From this description it is obvious that, in the mesentery of the whale at least, while a part of the chyle is poured out from the vasa inferentia into the proper cavities of the glands, another part of that fluid is made to circulate through the glands, entering them by the vasa inferentia, and leaving them by the vasa efferentia, without being poured into those cavities; and, from the close analogy which this animal bears to all others of the class of mammalia in what relates to the natural functions, as well as from similar bags being found in the horse and in man,—from all, in short, that we know of the structure and economy of these glands,—we must, I think, suppose the resemblance to hold in this respect also, rather than suppose that Nature

* Page 674-5.

would vary so widely in one or two minute particulars of a process, in the other parts of which she is so uniform. This is evidently Mr. Abernethy's opinion: for he says that the lacteals which pour the chyle into the bags are similar to those which terminate in the cells of the mesenteric glands of other animals. "There is also (he adds,) an analogy between the distribution of the lacteals in the inside of these bags, and that which we sometimes observe on the outside of the lymphatic glands in general. In either case, a certain number of the vasa inferentia, as they are termed, communicate with each other, and with the other vessels named vasa efferentia."

If this analogy be allowed with respect to the functions of these glands, it will likewise appear, from a careful perusal of Mr. Abernethy's memoir, that the portion of chyle which is poured into the glandular cavities by the vasa inferentia is taken up again, not by vasa lactea efferentia, but by red veins. He found numerous veins communicating with those cavities by open mouths, which could be for no other purpose but to receive something from them; and that something could only be the chyle which is poured into them by the vasa inferentia, mixed and combined doubtless with the peculiar secretion of the cavities. On the other hand, it does not appear that any direct communication exists between the cavities and the vasa lactea efferentia. Mr. Abernethy says, indeed, that, "when quicksilver was poured into any of the lacteals which were found near the sides of the bags, it immediately ran in a stream into their cavities;" and it is just possible that some of the lacteals, through which he thus injected the bags, may have been vasa efferentia: but, had this been the case, it seems more likely that the quicksilver which was thrown into them would have run on towards the root of the mesentery, than that it would have forced the valves, and taken a retrograde course into the bags. It seems at any rate highly probable that, had these been vasa efferentia, the same force which was sufficient to send the quicksilver back into the bags would have sent it also forward in the ordinary course of the vessels; but this does not appear to have happened. If it had, Mr. A. would certainly have mentioned it, as he mentioned the stream which flowed from the cut vessels at the root of the mesentery, when it was poured into those vasa inferentia which did not communicate with the proper cavities of the glands, but which he describes as communicating directly with the vasa efferentia. These are the only vasa efferentia of which he makes any mention, and, from his account of them, it is obvious that they have no direct communication with the glandular cavities. It is but fair to mention that Mr. A., in the paper now quoted, evidently takes it for granted that there are lacteals which do take their origin from these cavities, and carry off the chyle from them; but there is nothing in his narrative which, in my humble opinion, affords the slightest grounds for such an assumption; nor can I find any other grounds for it but the common, though equally gratuitous, assumption, that the whole of the chyle and lymph pass through the thoracic duct.

From the number of veins which Mr. Abernethy saw communicating by open mouths with the cavities in question, he concludes that a part of the chyle is taken up from them by those veins, and is thus introduced directly into the circulation. This conclusion is indeed so obvious, that I do not see how it can be questioned, or even doubted; and, if a part be taken up by veins, the whole may be so. If no other channel can be pointed out through which any part of the chyle gets from these glandular cavities into the venous system, we cannot avoid the conclusion that the whole is taken up by those "numerous veins" which have been so distinctly seen opening from the cavities, for no other conceivable purpose than that of absorbing their contents.

I have already stated my reasons for believing that the structure observed by Mr. Abernethy in the mesenteric glands of the whale, is not peculiar to that animal; that, on the contrary, there is a close analogy between the glandular bags described by him and the cells in the conglobate glands, as well as between the lacteals which pour chyle into the bags and those which terminate in the cells of ordinary conglobate glands; and that there is a similar analogy between the distribution of lacteals on the inside of the bags in the whale, and those convolutions of the same vessels which are met with in such numbers on the parietes of the cells of ordinary conglobate glands. In further proof of this analogy, and more especially as a proof that it holds not only with respect to the glandular cavities and the lacteal vessels, but likewise with respect to the absorption of chyle from those cavities by veins, I may mention that the idea of a communication between the lacteals and the vena portæ in man and other animals is by no means a recent invention; nor does it rest solely on opinion or reasoning from analogy. Nearly two hundred years ago, it was found, on tying the trunks of the lacteals, that the chyle passed into the vena portæ. Early in the last century, and repeatedly in the course of it, quicksilver, thrown into the absorbents of the stomach, intestines, and spleen, was observed to pass into the vena portæ;* and, although the existence of any such communication between the absorbents of the alimentary canal and the vena portæ† has been positively denied by some of the most respectable writers of a subsequent period, it seems now to be proved beyond doubt.

On the authority of Professors TIEDEMANN and GMELIN, we are informed that M. FOHMANN, dissector at the anatomical theatre of that university, and who has long been employed in examining the excretory glands of the intestinal canal and the absorbents, saw the quicksilver with which he had filled the lacteals of two seals force its way into the vena portæ; and that in subsequent experiments, performed with the greatest care and circumspection, in presence of Professors Tiedeman and Gmelin, on two dogs, one horse, one cow, and three human subjects, the injection (which in all was begun soon after death) reached the branches of the mesenteric veins and the vena portæ, without any external force being employed. On a closer examination, it turned out that the communication of the lacteals with the veins of the intestines took place in the mesenteric glands, and that all the veins proceeding from a gland which was filled with quicksilver also contained it.‡

It may be objected to the conclusions which have been drawn from these experiments, that the quicksilver may have found its way into the veins in consequence of rupture of the lacteals; but it seems very improbable that this fluid, when thrown thus gently into the lacteals, should not only rupture them, but the veins likewise, and then force its way into such flaccid vessels as the latter are known to be. It is very im-

* JOH. WALÆUS* found, on tying the trunks of lacteals, that the chyle passed into the vena portæ; ROSEN and WALLERIUS,† J. F. MECKEL,‡ J. F. LOBSTEIN,§ and G. C. LINDNER,§ perceived, on filling the absorbents of the stomach, of the intestinal canal, and of the spleen, with quicksilver, that it passed into the vena portæ; ASTLEY COOPER¶ also found quicksilver in the vena portæ, after injecting with it the absorbents of the mesentery. (Edinb. Med. and Surg. Journ. xvii. 462-3.)

† HALLER, MASCAGNE, CRUICKSHANK, LIEUTAUD, PORTAL, SOEMMERRING, HEWSON, and others. (Ibid. 463.)

‡ Edinburgh Med. and Surg. Journal, xvii. 463.

---

* Epistolæ duæ de Motu Chyli et Sanguinis ad Th. BARTHOLINUM, in Bartholini Anatomia, edit. 5. p. 7, 8, 9.

† De Excitentia Vasorum Absorbentium in Intestinis.—Upsal, 1731.

‡ Nova Experimenta et Observationes de finibus Venarum ac Vasorum Lymphaticorum.—Berlin, 1772; p. 5.

§ De Liene.—Argentorati, 1774.

|| De Lymphaticorum Systematc.—Hal. 1787-8.

¶ Med. Records and Researches selected from the Papers of a Private Medical Association.—Lond. 1798; vol. i.

probable that this should happen in any one instance, even if the quicksilver were thrown into the lacteals with considerable force. It is still more unlikely to have happened in every one of the seven bodies that were subjected to the experiment, and more especially so as we are assured that no external force was employed.*

These experiments of M. Fohmann, and the observations of Mr. Abernethy on the mesenteric glands of the whale, seem mutually to corroborate and illustrate each other. Fohmann's experiments, together with the casual observations referred to as made by others, of both chyle and quicksilver passing from the lacteals to the branches of the vena portæ, seem to put it beyond a doubt that the structure observed by Mr. Abernethy in the whale exists also, with certain modifications, in man and many other animals; and that, in the latter as well as in the former, a part of the chyle is poured by the vasa lactea inferentia into certain bags, or cells, in the substance of the mesenteric glands, from whence it is taken up by the extremities of veins, and carried directly into the circulation. On the other hand, Mr. Abernethy's observations on the mesenteric glands of the whale, of the horse, and of man, enable us to form a distinct idea of the mode in which the communication observed by those other anatomists is probably effected in the glands. M. Fohmann's experiments seem to prove that the veins *do* receive chyle from the lacteals in the mesenteric glands; while Mr. Abernethy's observations and experiments point out the *mode* in which they probably do so.

As a farther evidence of the truth of these observations, I may mention that, from the time anatomists became acquainted with the lymphatic system, many men of the first rank in the profession have considered the thoracic duct as quite incapable of transmitting the whole of the chyle and lymph, even with the aid of those other lymphatic trunks that are found to anastomose with it. If these fluids, then, cannot all pass into the blood through the trunks of the lymphatic system, a part must find some other channel; and if the view I have now endeavoured to give of the interior structure and economy of the conglobate glands be at all correct, these would seem to afford such a channel. It has, indeed, been maintained by many, that the whole of the fluids in question may and do pass through either the thoracic duct itself, or some of the other lymphatic trunks anastomosing with it, and leading into the great veins near the heart; but

* Edinburgh Med. and Surg. Journal, xvii. 463.

the diameters of all these trunks taken together bear no just proportion either to the size and number of the vessels which constitute the rest of the lacteal and lymphatic systems, or to the quantities of fluid which we have every reason to believe that this system occasionally transmits in a given time. Boerhaave, for instance, mentions a man who drank sixteen pints of wine daily; Haller gives examples of persons drinking two hundred ounces of mineral waters in a few hours, and passing the whole by urine;* and, from what I have repeatedly heard of the feats performed every season with some of our own mineral waters, I see no reason to doubt the accuracy of these statements. These are quantities which we cannot suppose to pass through tubes of so small diameter in so short a time. It is, indeed, contended that the velocity with which fluids pass through the thoracic duct may be such as to account for its transmitting even these quantities in the time mentioned; and, in support of this opinion, Mr. Cruickshank mentions that the chyle in the lacteals of the mesentery of dogs, in some of his experiments, evidently ran through a space of four inches in a second of time, which is twenty feet in a minute. This would seem, however, to have been but a transient acceleration of its motion, caused probably by the sudden admission of cold air, or by some other irritation attendant upon the experiments: and, on the other hand, some experiments of M. Magendie's gave a very different idea of the velocity with which the chyle passes through the thoracic duct. Upon opening this duct in the neck of an ordinary sized living dog, which had a little before eaten as much animal food as he chose, M. Magendie found the chyle discharged at the rate of about half an ounce in five minutes, or six ounces in an hour; and he says it continued to discharge at this rate as long as digestion continued, which was for a good many hours.† In man, whose organs are on somewhat a larger scale, the quantity of chyle passing through the thoracic duct may be proportionally greater; but to suppose that, in the case mentioned by Haller, two hundred ounces of fluid passed through it in a few hours,—let us even allow six hours,—would be to assign it a rate of above thirty-three ounces in the hour, instead of six or seven, as Magendie's experiments would lead us to expect. Such velocity is perhaps possible. Mr. Cruickshank's observation obliges us to allow that it is so, at least for a few seconds at a time; but it is very impro-

* Cruickshank on the Absorbents, p. 30.

† Precis Element. ii. 164.

bable that the thoracic duct ever does transmit the quantities of fluid I have mentioned with such velocity, or even that it could do so; especially if we allow that it has, over and above, to transmit the ordinary current of lymph, which is supposed to be constantly passing through it in greater or less quantity. We must therefore suppose that there is some other channel through which matter may pass from the alimentary canal to the sanguiferous system; and (I repeat) the experiments and observations now mentioned seem to show that, whatever other channels there may be, one is through the mesenteric glands.

Anatomists describe a peculiar fluid as occasionally found in the cellular substance of the conglobate glands, principally in young animals, generally of a whitish colour, sometimes inclining to red, sometimes blue, and occasionally almost black. This fluid, when viewed in the microscope, appears to have globules similar to those found in milk, and has generally been distinguished by the name of the succus proprius glandularum of Haller. It is described as totally different from the absorbed fluid passing through the glands, and is probably a peculiar secretion. It is said to be found in the common cellular membrane which connects together the vessels, nerves, and proper cells of the gland, but authors do not seem to be precise in this part of their description. Haller, who is the authority commonly referred to with respect to this fluid, does not seem to have had a very definite idea of the cellular structure of the conglobate glands, and could not therefore determine the precise seat of this secretion. Indeed, when we find one of the most zealous and inquisitive physiologists of the present day speaking of the fluid as obtained by squeezing the gland between the fingers,* we may conclude that the point in question has not yet been minutely investigated. As we do not find the common cellular membrane endowed with the property of secreting peculiar fluids in any other part of the body, it would seem much more probable that this is secreted by the proper glandular cells. It seems evident, at least, from the foregoing observations and experiments, that there is a peculiar fluid secreted in these cells, and that this secretion is intended to be mixed with, and to act upon, the absorbed fluids which are poured into them by the vasa inferentia, in order to produce in these absorbed fluids certain changes, by which they may be fitted for becoming a part of the circulating mass of blood. There is, I think, equal reason to believe that a similar, or at

* Magendie, Precis Element. tome ii. 59.

least an analogous, change is effected in the other portion of the absorbed fluids, which I mentioned as pervading the gland without being poured into its cells,—entering by the vasa inferentia, and leaving it by the vasa efferentia. It is not, indeed, so easy to conceive how the change is effected on this latter portion of the absorbed fluids, as on that which is poured into the proper cells of the gland, and there actually mixed with the peculiar secretions of those cells; but we know, from the analogy of other functions, that a circulating fluid may undergo very important changes in certain situations, without leaving the vessels in which it circulates, and therefore without coming in actual contact—certainly without mixing—with the fluid or fluids which we suppose to be chiefly instrumental in producing those changes. In respiration, for instance, all agree in attributing very important changes in the condition of the blood to the agency of atmospheric air in the lungs; yet no one contends that the blood is poured into the air-cells, or that it is in any way mixed or brought into actual contact with the atmospheric air. On the contrary, it is the universal opinion of physiologists,—and may be said to be demonstrable,—that it passes directly from the terminations of the pulmonary artery into the beginnings of the pulmonary veins. In like manner the chyle and lymph, circulating through the substance of a conglobate gland, may be acted upon by the secretions of that gland, without mixing or coming in contact with those secretions.

Anatomical observations have shown that the lacteal and lymphatic vessels pervade the glands to an extreme degree of minuteness. The contents of these vessels have therefore, as far as we can see, the same opportunity of intercourse with the secretions of the glands, as the blood in the pulmonary artery has with the air in the cells of the lungs; and the ramifications of the lacteal and lymphatic vessels on the parietes of the glandular cells, appear so analogous to those of the pulmonary artery on the parietes of the air-cells of the lungs, that the analogy most probably extends also to the effects produced on the fluids circulating in the ramifying vessels. If so, the chyle and lymph circulating on the parietes of the glandular cells may be regarded as influenced and altered by the contents of those cells, in a manner analogous to that in which the blood is by the air in the lungs. Of the particular nature of the influence exerted, or the alteration effected in either case, we know little or nothing; but, if we have reason to believe that the blood owes its change of state in respiration principally to the discharge of certain matter by the exhalent extremities of the pulmonary artery, a similar conclusion seems to be equally deducible with respect to the chyle and lymph in the conglobate glands, from what we know of the structure and economy both of these glands and of the lymphatic system in general. It is obvious from Mr. Abernethy's paper, that the vasa inferentia, after discharging a part of their contents into the proper glandular cavities, transmit the remainder to the vasa efferentia, in the same way (for aught we know) as the pulmonary artery transmits the blood to the pulmonary veins; and, although we cannot demonstrate such obvious difference between the contents of the vasa-lactea inferentia and efferentia, as between the blood of the pulmonary artery and veins, we have reason to believe that both the chyle and lymph do undergo important changes in the glands, and that these changes are not confined to a part of the fluids in question, but affect the whole, more or less. It seems, therefore, but reasonable to suppose that the separation of parts which these fluids have been shown to undergo in the glands, is immediately subservient to those important changes, and probably one of the essential means of effecting them; as the separation of the carbon, &c. from the blood in the lungs is of effecting the changes we see this fluid undergo in respiration.

*Postscript.*—I have received the last Number of your Journal, containing Dr. Rossi's "Remarks on the Communication of the Lymphatic Vessels with the Veins." These remarks, as far as they go, appear to me to confirm very decidedly both the observations of Mr. Abernethy,* and the conclusions drawn from them in the paper I sent you some time ago, "on the Internal Structure and Economy of the Conglobate Glands:" the more so as Dr. Rossi does not seem to be acquainted with Mr. Abernethy's paper. Had he ever seen that paper, or been aware of its contents, he would not have said that "the argument (for the existence of the communication) is only upheld by the passage which the mercury makes into the venous ramifications;" and that, "although the injection evidently shows this passage, yet it may not exist in nature, because it may be owing to the impetus of the injection."† His subjoined dissection of the inguinal glands and lymphatics, in a case of anasarca of the lower extremities, need not, in my humble opinion, shake our belief in the existence of the communication in question. If the view of the subject which I have attempted to give be at all correct, we need not be surprised that Dr. Rossi did not detect lymph in the small veins passing out of the glands, mixed (as it must have been) with the blood of those veins, and altered or assimilated to blood (as it most probably had been) by the process it had previously undergone in the cells of the glands.

*Aberdeen; 5th January,* 1827.

* Phil. Trans. anno 1796. † P. 83.

